# Comparison of Pocock and Simon’s covariate-adaptive randomization procedures in clinical trials

**DOI:** 10.1186/s12874-024-02151-3

**Published:** 2024-01-25

**Authors:** Guogen Shan, Yulin Li, Xinlin Lu, Yahui Zhang, Samuel S. Wu

**Affiliations:** Department of Biostatistics, University of Florida, Gainesville, 32610 FL USA

**Keywords:** Additional covariates, Allocation predictability, Covariate adaptive randomization, Imbalance score, Pocock and Simon, Statistical power

## Abstract

When multiple influential covariates need to be balanced during a clinical trial, stratified blocked randomization and covariate-adaptive randomization procedures are frequently used in trials to prevent bias and enhance the validity of data analysis results. The latter approach is increasingly used in practice for a study with multiple covariates and limited sample sizes. Among a group of these approaches, the covariate-adaptive procedures proposed by Pocock and Simon are straightforward to be utilized in practice. We aim to investigate the optimal design parameters for the patient treatment assignment probability of their developed three methods. In addition, we seek to answer the question related to the randomization performance when additional covariates are added to the existing randomization procedure. We conducted extensive simulation studies to address these practically important questions.

## Introduction

Randomized controlled trials (RCTs) are critical in evaluating the effectiveness of new treatments and interventions in clinical research. Randomization is a fundamental element of clinical trials, primarily aimed at preventing selection bias and enhancing the validity and accuracy of the results [1–4]. When sample size is large enough, influential factors are more likely to be balanced approximately. However, in the case of a large number of influential factors, complete randomization (CR) carries a substantial risk of chance imbalance in these factors [5]. For trials with limited sample sizes, CR can lead to significant disparities in the number of participants across groups [6–8]. Such chance imbalances in baseline covariates and group sample sizes can ultimately undermine the statistical power and may present challenges in comparing treatment groups and interpreting trial results. For instance, in clinical trials involving relatively small sample sizes, simple randomization methods have been shown to produce an unequal distribution of both participants and influential factors across treatment and control groups [9, 10].

Stratified block randomization (SBR) is frequently employed to mitigate imbalances in baseline covariates across groups. In a review article by Lin et al. [3], they found that SBR designs were used in the majority of trials (close to 70%). However, its capability is constrained to a limited array of factors. With an extensive number of strata, the efficacy of SBR in maintaining balance among treatment groups can be compromised as some strata may end up with very few participants [5, 10, 11]. When the global treatment balance is the target of a randomization procedure, some existing designs may be utilized, such as the permuted block design (PBD) [12], Efron’s biased coin design [13], Wei’s urn design [14]. Over recent decades, various innovative restricted randomization designs have been developed, building upon the biased coin design and the urn design. These include the big stick design, the biased coin design with imbalance tolerance, the Ehrenfest urn design and the block urn design [15–18]. These recent methods have demonstrated better performance than traditional methods, particularly in maintaining a delicate balance between imbalance score and allocation predictability.

Covariate adaptive randomization (CAR) is a popular minimization method to achieve balance over a broader spectrum of covariates [19–25]. The concept of CAR approach was first introduced with the focus on dynamically identifying the treatment that could minimize the overall imbalance across covariates [23]. Pocock and Simon later proposed a generalized and flexible CAR approach [26]. One of the primary features of their method is the incorporation of allocation probabilities [4, 27–29]. After temporarily assigning a newly recruited participant to all the available groups, each group ends up with its own total number of imbalances. Then, instead of strictly assigning the new participant to the group with lowest total number of imbalances, the participant will be allocated to that group with a probability [22]. This means that while the option that would minimize imbalance is weighted more heavily, there is still a chance that the participant could be allocated to other groups to improve randomness. The employment of the probabilistic element introduces another layer of randomization, which can be beneficial for reducing bias and confounding [30–33]. Another established method for achieving covariate balance is the dynamic hierarchical randomization which offers an alternative to minimization when there are too many stratification factors. Such approaches not only maintain randomness but also ensure balance across each covariate level, accommodating varying degrees of imbalance among different covariates [34].

In this article, we critically examine several research questions emerging from the application of Pocock and Simon’s (PS) method in ongoing clinical trials [35]. First, Pocock and Simon proposed three methods to determine the treatment assignment probability after each new patient. A pivotal aspect of our research is to identify the optimal parameters within these methods that yield the best possible performance. Furthermore, our study delves into the exploration of the impact on the randomization method performance when adding new prognostic factors to the CAR procedure. Specifically, we aim to explore to which extent the overall performance can be enhanced through the incorporation of different number of factors. This will allow us to ascertain whether the inclusion of more factors leads to increasingly accurate and reliable results, or if there are diminishing returns beyond a certain point. In addition, we proactively consider increasing the number of study sites to mitigate the risks associated with low patient recruitment rates. Lastly, our study also conduct a comparative analysis of statistical power between CR and PS designs.

The structure of this article is outlined as follows. In Methods section, we illustrate Pocock and Simon’s minimization method. We describe the three formulas used for calculating treatment assignment probabilities, the calculation of treatment imbalance score and allocation predictability that evaluate randomization procedures. Then, in Numerical study section, we first run simulation studies to address the three research questions mentioned above. After that, we use data from a trial for patients with pleural infection to demonstrate the application of CAR designs and further examine our research questions. In Discussion section, we provide some comments about potential topics we can explore in the future.

## Methods

For a study with *K* treatments, suppose there are *I* prognostic factors that need to be balanced to assess the treatment effect properly. We consider categorical prognostic factors in this article. Suppose $J_i$ is the total possible level of the *i*-th prognostic factor, where $i=1, 2, \cdots , I$. For the *i*-th prognostic factor, let $X_i$ be the level value: $X_i \in \{1,2, \cdots , J_i\}$. One example is the disease severity with three levels as mild, moderate, and severe.

When the *i*-th factor is considered, the number of already enrolled participants can be organized in a *K* by $J_i$ contingency table. Suppose $N_{k,x_i|i}$ is the number of participants being assigned to treatment *k* whose *i*-th factor value is $x_i$. Then, $\sum \nolimits _{k=1}^K \sum \nolimits _{x_i=1}^{J_i} N_{k,x_i|i}$ is the sum of all numbers from the *K* by $J_i$ table, and that is the total enrolled participants so far.

When a new participant is recruited for this study, that participant is ready for randomization after collecting the information of the *I* prognostic factors: $\{x_1,x_2, \cdots , x_I\}$. If treatment *k* is assigned to this new participant, $N_{k,x_i|i}$ is increased by 1, and other values in the *K* by $J_i$ table for the *i*-th factor remain the same. The values at the $x_i$-th column of the *K* by $J_i$ table become

$$\begin{aligned} Y(x_i|i,k)=(N_{1,x_i|i},\cdots , N_{k-1,x_i|i},N_{k,x_i|i}+1, N_{k+1,x_i|i}, \cdots , N_{K,x_i|i}). \end{aligned}$$

These numbers are then used to calculate the imbalance value for the *i*-th factor after adding one more participant to treatment *k*. Following Pocock and Simon [26], that imbalance value is denoted as $d_{ik}=D(Y(x_i|i,k))$, where *D* is a function to calculate the imbalance value. In practice, three methods based on the range, variance, and standard deviation of $Y(x_i|i,k)$ can be used, and we refer them to be as the range method, the var method, and the SD method to compute the imbalance value. A total of *I* imbalance values $d_{ik}$ $(i=1, 2, \cdots , I)$, can be calculated by using one of the three methods. These imbalance values are then used to calculate the total imbalance score for assigning this new participant to treatment *k*. These individual imbalance values can be combined in multiple approaches to obtain the total score. We consider an equal weight in calculating the total imbalance score during the randomization procedure

$$\begin{aligned} S_k=\sum \limits _{i=1}^{I} d_{ik}. \end{aligned}$$

These total imbalance scores: $S_k$ $(k=1,2,\cdots ,K)$, are sorted from the smallest to the largest, with the associated probabilities $p_1$ to $p_K$, where $p_1\ge p_2\ge ...\ge p_K$, and $\textbf{P}=\{p_1,p_2, \cdots , p_K\}$. A treatment having a small value of imbalance score has a high probability in the treatment assignment. It should be noted that the computed imbalance score $S_k$ is the covariate imbalance score.

### Treatment assignment probability

Three formulas for $\textbf{P}$ were proposed by Pocock and Simon [26]. The first two formulas were developed by using the ordering of $S_k$ values. The first one is relatively simple

$$\begin{aligned} p_1=p \ \ \text {and}\ p_{k}=\frac{1-p}{K-1}, \ k=2, 3, \cdots , K. \end{aligned}$$

To reduce the overall imbalance score, *p* should be larger than 1/*K*. In the illustrated example in Pocock and Simon [26], for a study with 3 treatments, *p* was chosen to be 2/3. Once the value of *p* is chosen, the treatment assignment probabilities are determined. We refer to this approach as the PS_p_ approach. The second formula is

$$\begin{aligned} p_k = q - \frac{2(Kq-1)}{K(K+1)}k, \end{aligned}$$

where $k=1,\cdots ,K$, and *q* is a constant between 1/*K* and $2/(K-1)$. We refer to this as the PS_q_ approach.

In addition to the ordering of $S_k$ values, the third formula used the $S_k$ values in computing the treatment assignment probabilities as

$$\begin{aligned} p_k = \frac{1}{K-t}\left[ 1 - \frac{tS_k}{\sum S_k}\right] ,\,\ \,k=1,\cdots , K, \end{aligned}$$

where *t* is a constant between 0 and 1. We refer to this approach as the PS_t_ approach. The last formula is relatively more complicated than the first two formulas. Once the value of *p* in PS_p_ or *q* in PS_q_ is chosen, the probabilities are determined. However, in the last formula, the probabilities could be changed as the values of $S_k$ are updated after each new patient.

In general, the PSp approach assigns the treatment arm with the minimal total imbalance score a probability higher than the mean, while the remaining arms equally share the leftover probability; the PSq approach distributes probabilities in a monotonically decreasing pattern based on the rank of the total imbalance score with a constant decrement value; the PSt approach assigns treatment probabilities by inversely weighting them against the total imbalance scores for each arm and dynamically updates the probabilities with each new patient.

In the simulation studies, we included the following randomization methods: CR, stratified block randomization, the stratified big stick design (SBSD) by Zhao [25], and the hierarchical dynamic balancing randomization (HDBR) by Heritier et al. [36]. The SBSD design is a two-stage CAR randomization method that can improve balance and randomness of a trial as compared to the traditional stratified permuted block randomization. The HDBR design is a dynamic balancing randomization with the constraint on the importance ordering of factors [36]. The R codes can be downloand from the GitHub page: https://github.com/AdaptiveDesign/CAR_randomization.

### Treatment balance and allocation predictability

In randomized clinical trials, treatment balance and allocation predictability are two essential metrics used to evaluate the performance of randomization procedures. These two values are traditionally calculated at the end of the trial, after all participants have been randomized. Suppose $N_k(m)$ is the number of participants being assigned to treatment *k* after *m* patients enrolled, where $k = 1,\cdots , K$ and $0\le N_k(m)\le m$. Then, we have $\sum \nolimits _{k=1}^K N_k(m)=m$. In order to calculate the final treatment imbalance score, we begin by applying the range method [37] to compute the imbalance value following the enrollment of each individual patient

$$\begin{aligned} D(m) = \max (\left\{ N_k(m)\right\} _{k=1}^K) - \min (\left\{ N_k(m)\right\} _{k=1}^K), \end{aligned}$$

where $m=1, 2, \cdots , N$. Following the literature [38–40], we utilized the treatment imbalance score (IS) as

$$\begin{aligned} IS = \frac{1}{N} \sum \limits _{m=1}^N \frac{D(m)^2}{m}. \end{aligned}$$

The value of imbalance score represents the lost information [40]. A randomization procedure with a low value of imbalance score is preferable. The treatment imbalance score, IS, is used to compare different randomization methods including the CR in which there is no covariate involved.

Allocation predictability (AP) is the probability of accurately predicting treatment assignments from the calculated imbalance score, under the assumption that the investigator consistently predicts the treatment with the higher likelihood of allocation [11, 36]. AP is a commonly used metric for assessing the lack of randomness of an allocation process, with a lower AP indicating a more unpredictable randomization procedure [8]. When the *m*-th participant is assigned to the treatment group with the lowest imbalance score, the guess is correct, denoted as $G_m = 1$. Otherwise $G_m = 0$ for wrong guess. For a two-arm study designed with complete randomization, the probability of $P(G_m = 1)$ is 50%. For a study with *K* treatments, allocation predictability can be defined as

$$\begin{aligned} AP = \left( \frac{1}{N}\sum \limits _{m=1}^N P(G_m = 1) -\frac{1}{K}\right) \frac{K}{K-1}. \end{aligned}$$

The range of $\frac{1}{N}\sum \nolimits _{m=1}^N P(G_m = 1)$ is from 0 to 1. The quantity $\frac{K}{K-1}$ is added to the AP calculation to make sure that upper limit of AP is 1. For a CR design, the AP value will be very close 0. Thus, it is preferable to have a randomization procedure with a low value of AP^2^.

In the simulation studies, we used rescaled IS and AP value (IS_r_ and AP_r_) to calculate the weighted score for comparing different methods. The weighted score is defined as

$$\begin{aligned} \Omega = \sqrt{\frac{IS_r^2 + AP_r^2}{2}}. \end{aligned}$$

When multiple values of the parameter (e.g., *p* in the PS_p_ method) are studied, the computed IS values are rescaled to 0 and 1 as the IS_r_ values. The AP_r_ values are calculated by using AP values in a similar approach. A randomization procedure having a small weighted score $\Omega$ is preferable.

## Numerical study

We first run simulation studies to identify the optimal parameter of the three treatment assignment methods (PS_p_, PS_q_, and PS_t_) having a good performance with regard to IS, AP and the weighted score $\Omega$ [41].

### Optimal parameter in the PS randomization procedures

For a study with 3 treatments, let sample sizes per arm be: $n=5, 10 ,20, 30, 50, 80$, and 100. Two influential factors are considered: factor 1 that follows a Bernoulli distribution with $p=60\%$, and factor 2 following a multinomial distribution with three possible outcomes with probabilities of (30%, 20%, 50%).

**Fig. 1:**
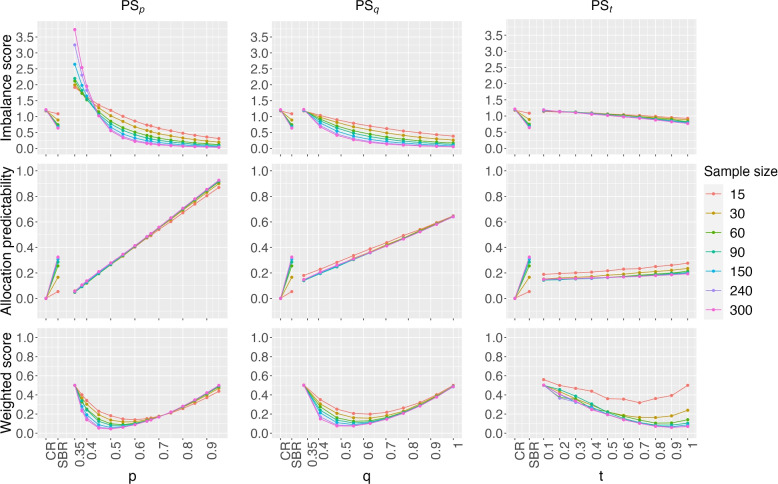
Imbalance score, allocation predictability, and weighted score of the PS procedure based on three treatment assignment probability methods: PS_p_, PS_q_, and PS_t_. These methods are compared with CR, and SBR with the block size of 6 for a study with 3 treatments and two influential factors

Figure 1 shows the comparison between the three treatment assignment methods based on the range approach to calculate the covariate imbalance score. We used a block size of 6 in the SBR in this figure. For each given sample size, SBR could have a lower imbalance score than CR while SBR has larger AP values than CR. In the PS_p_ method, the parameter *p* is from 0.35 to 0.95 (left side). Imbalance score decreases as *p* goes up for each given sample size. As *p* gets large, the probability of a correct guess on treatment assignment becomes high. For that reason, allocation predictability is an increasing function of *p*. For weighted score $\Omega$ considering both rescaled imbalance and allocation predictability, the optimal *p* is found to be between 0.4 and 0.6 to have a good balance between imbalance and allocation predictability. When sample size is small (e.g., 15 per arm), the optimal *p* is near 0.6. That optimal value is reduced to 0.5 when $n>100$ per arm. In Fig. 1, we observed similar results when PS_q_ was used to define the probability of treatment assignment. The optimal *q* value is close to 0.60 when n$<100$, and that value is reduced to 0.5 when $n>100$ as observed. For the PS_t_ method, the weighted score is larger than other methods when sample size is very small (e.g., 15 in a study). In other cases, the PS_t_ method has good performance as the computed IS and AP values are almost independent of the choosing *t* values. In Fig. 1, we found that *t* near 0.8 provide a good balance between IS and AP for the PS_t_ method.

The findings using other approaches (e.g., the var approach, and the SD approach) for the covariate imbalance score calculation, are similar to those observed in Fig. 1. In general, the SD approach has lower $\Omega$ values than the var approach. The SD approach considers the sample sizes in the imbalance score calculation. Based on that finding, we focus our comparisons on the range approach and the SD approach in computing the covariate imbalance score ($S_k$) in the PS methods to determine the treatment assignment during the study. Once the study is completed, the treatment imbalance score IS is computed to compare different methods regarding treatment imbalance.

**Fig. 2:**
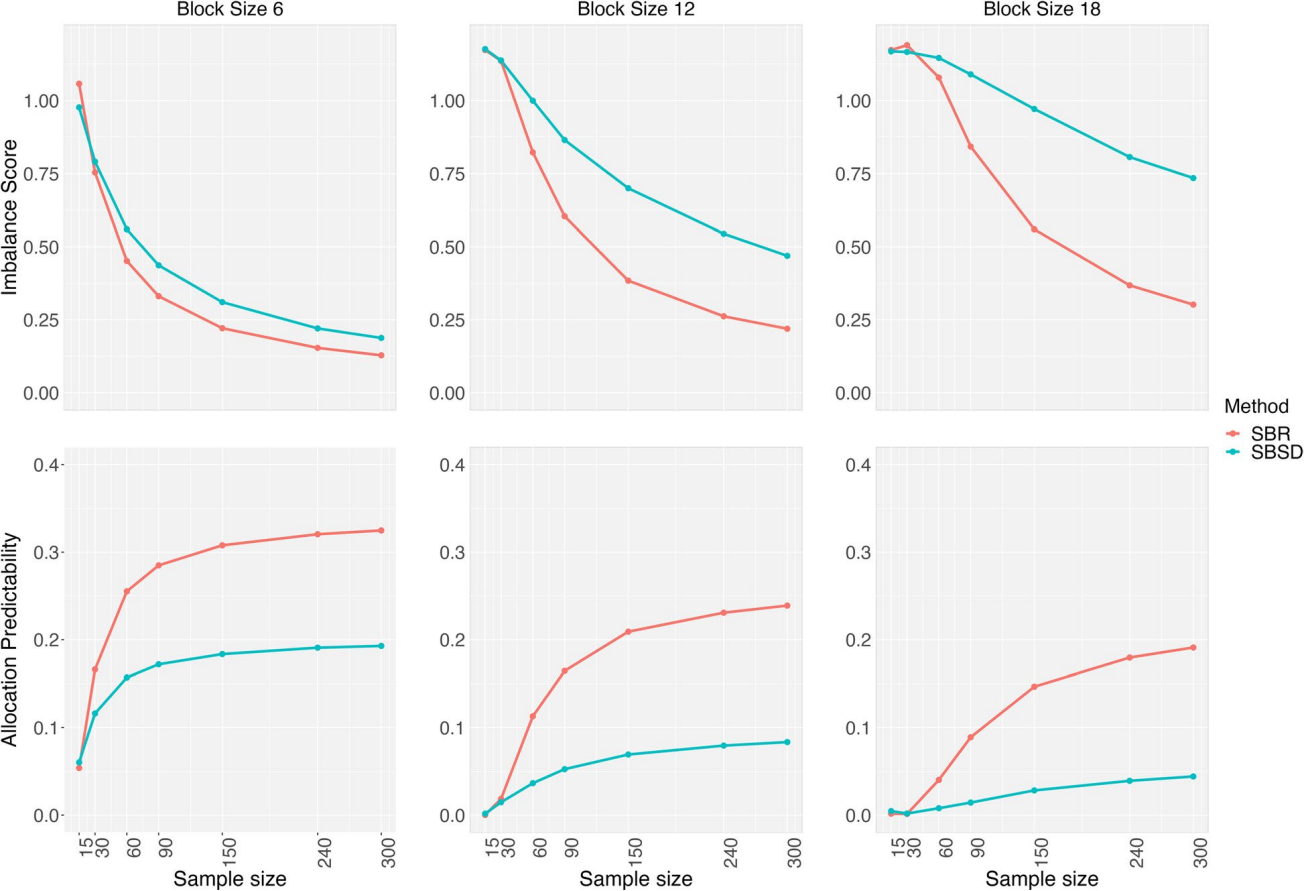
Imbalance score and allocation predictability of the SBR and SBSD methods with the block sizes of 6, 12, and 18. SBR: stratified block randomization; SBSD: stratified big stick design

In Fig. 1, we study the SBR method with the block size of 6. We further compare the SBR method with the block sizes of 6, 12, and 18, and include the SBSD method in Fig. 2 for comparison. The sample size is the same as that in Fig. 1. For the two methods, the IS value is a decreasing function of sample size, while that trend is reversed for the AP value. The SBR design has lower IS values than the SBSD method, and their difference gets larger as the block size goes up from 6 to 18. However, the SBSD method can improve the randomness of a trial by reducing a constant AP value for each given sample size. For a study with the sample size of 300, their difference in the AP value is close to 0.14 from these three plots.

**Fig. 3:**
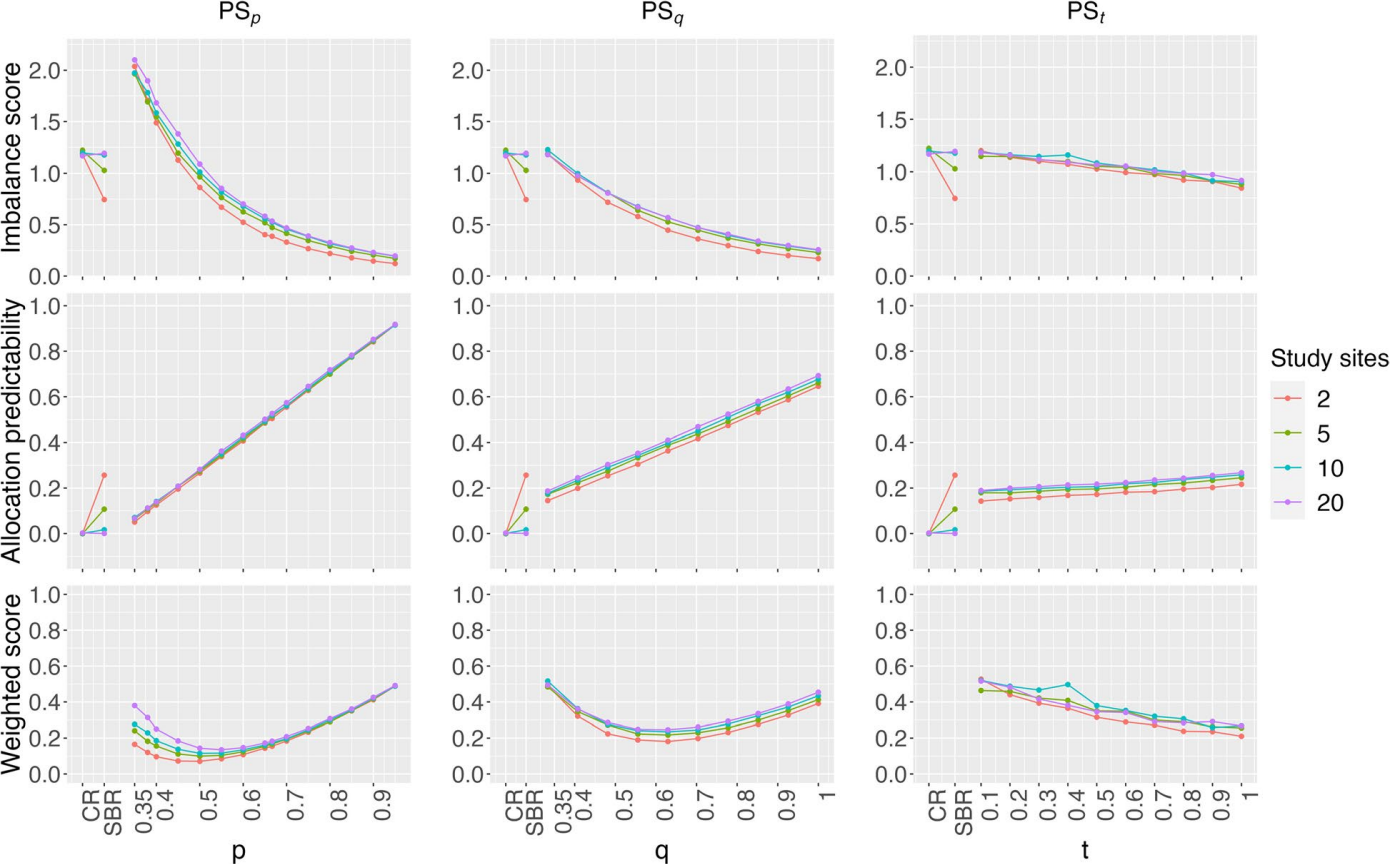
For a 3-arm study with the total sample size of 60, imbalance score, allocation predictability, and weighted score are plotted as a function of the parameters in the three PS methods, when the number of study sites is increased from 2 to 20

We also studied the effect of the number of study sites on weighted score in Fig. 3, with the first factor as the number of study sites, and the second factor follows a multinomial distribution with three possible outcomes with probabilities of (30%, 20%, 50%). In general, imbalance score is a decreasing function of *p* or *q* in the PS_p_ or PS_q_ method, while that trend is reversed for allocation predictability. A study with a large number of study sites (e.g., 20 study sites) often has worse weighted score $\Omega$ than studies with less number of sites. The weighted score decreases more when the number of study sites is increased from 2 to 5, than the cases when the number of study sites is increased from 5 to 10 or from 10 to 20.

### Additional factors in CAR procedures

**Fig. 4:**
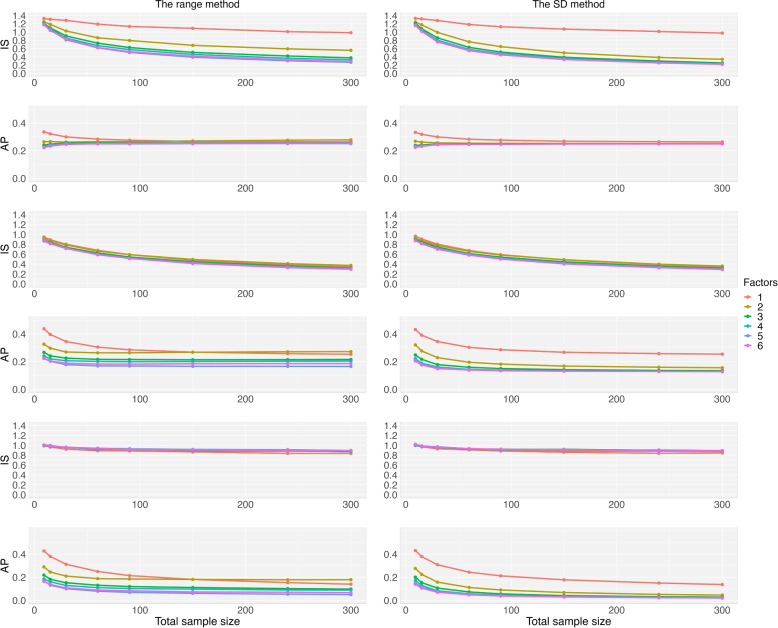
Imbalance score and allocation predictability of the three PS methods: the PS_p=0.5_ method (rows 1 and 2), the PS_q=0.5_ method (rows 3 and 4), and the PS_t=0.8_ method (rows 5 and 6), as a function of the total sample size and the number factors in the CAR for a study with 3 treatments. The range approach (left) and the SD approach (right) are used in the covariate imbalance score calculation

Another research question we tried to address in this article is to investigate the IS and AP performance when additional prognostic factors are added to CAR procedures. In Fig. 4, we presented the IS and AP values as a function of the total sample based on the range method (left) and the SD method (right) in calculating the covariate imbalance score $S_k$ for a study with 3 treatments: the PS_p=0.5_ method (rows 1 and 2), the PS_q=0.5_ method (rows 3 and 4), and the PS_t=0.8_ method (rows 5 and 6). Here, PS_p=0.5_ has the probabilities as $(p_1,p_2,p_3)=(50\%, 25\%, 25\%)$ while $(p_1,p_2,p_3)=(42\%, 33\%, 25\%)$ in PS_q=0.5_. In the PS_t=0.8_ method, the probability $(p_1,p_2,p_3)$ is not equal for each new patient. It can be seen that as compared to PS_q=0.63_, PS_p=0.6_ has a higher probability to assign a new patient to the treatment having the lowest imbalance score. In addition to the two aforementioned influential factors (one binary, one multinominal with three levels), we added another four factors having 2 or 3 levels, with a total of 6 factors.

In Fig. 4, we found that the IS values (rows 1, 3, and 5) decrease as sample size goes up. The curves are relatively flat for the PS_t_ method as compared to the other two equal allocation methods. When sample size is not too small with more than 1 factor, the two equal allocation methods have smaller IS values than the PS_t_ method. However, the PS_t_ method has lower AP values than the other two methods. When sample size is 300, the average of the IS values are 0.56 and 0.52 for the range method and the SD method, respectively. We also found that as compared to the range method, the SD method can reduce the average of AP values by 20% from these data. The SD method generally has better performance than the range method.

**Fig. 5:**
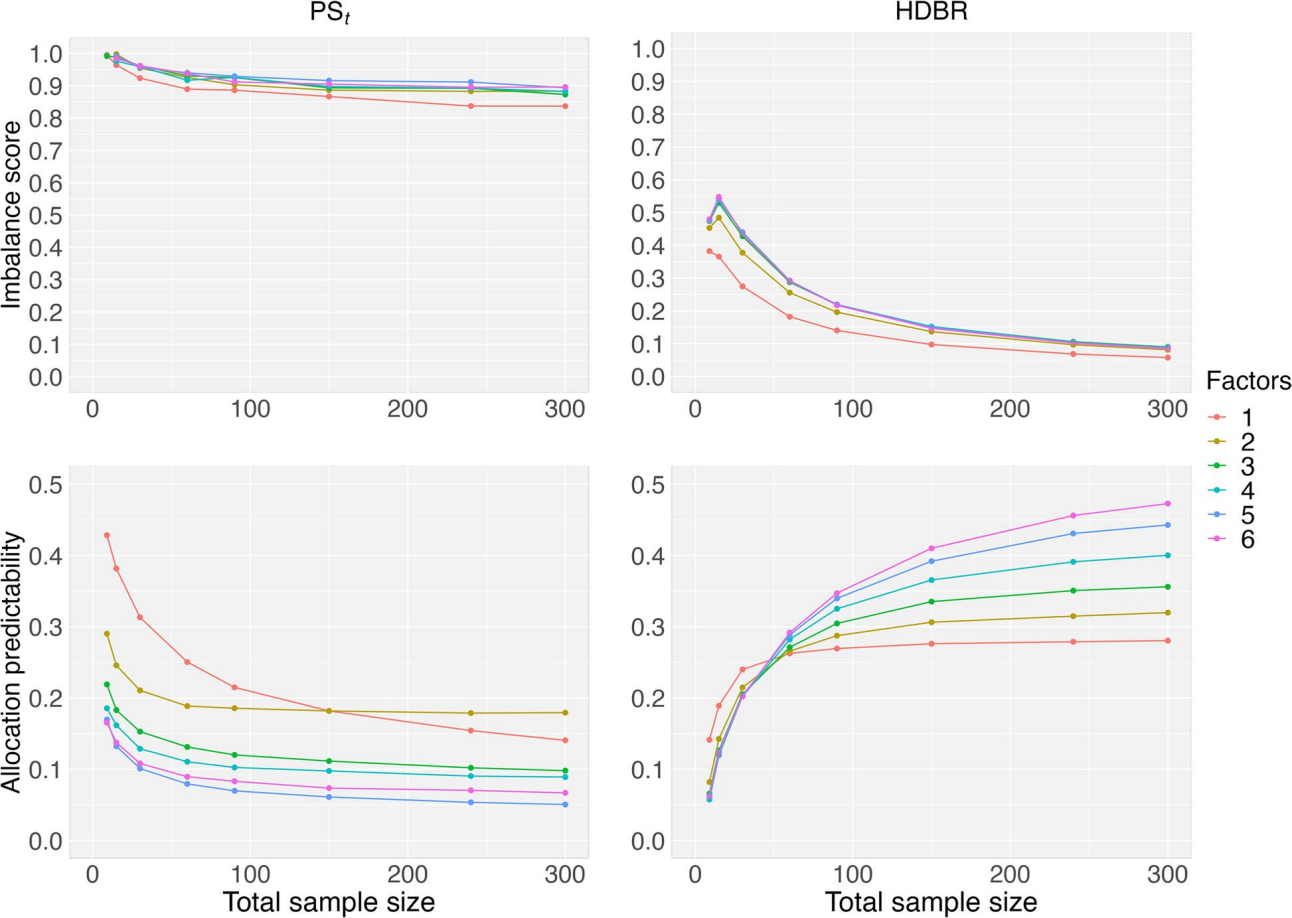
Imbalance score and allocation predictability of the PS_t_ method and the HDBR method as a function of the total sample size and the number factors in the CAR for a study with 3 treatments. HDBR: hierarchical dynamic balancing randomization

We further compared the PS_t_ method and the HDBR design in Fig. 5 with regards to IS and AP as the number of factors increases when the range method was used in the covariate imbalance score calculation. The HDBR design by Heritier et al. [36] aimed to maintain the marginal balance over important factors. It is expected that the HDBR design can reduce the IS values as compared to the PS method. The randomness index AP increases as sample sizes go up for the HDBR design. When sample size is 100 or above, the AP values of the HDBR design are larger than those for the PS_t_ method. If a low IS value is more important than the AP value, the HDBR design is a great randomization method to be utilized. Otherwise, the PS_t_ method could be utilized to have low AP values.

### Statistical power comparison

We compared the statistical power between CR and the CAR designs with PS_p=0.6_ with the sample size of 200 per arm in a two-arm randomized trial with the expected effect size of $0.4/\sqrt{2}=0.28$. The sample size was determined to detect the effect size of 0.28 to achieve the statistical power of 80% when $\alpha =0.05$. Suppose the first two factors are: binomial with *p*=50%, and multinomial with 3 outcomes having the equal probability. The correlation between the binomial factor and the outcome is $\rho _1=$0.3.

**Fig. 6:**
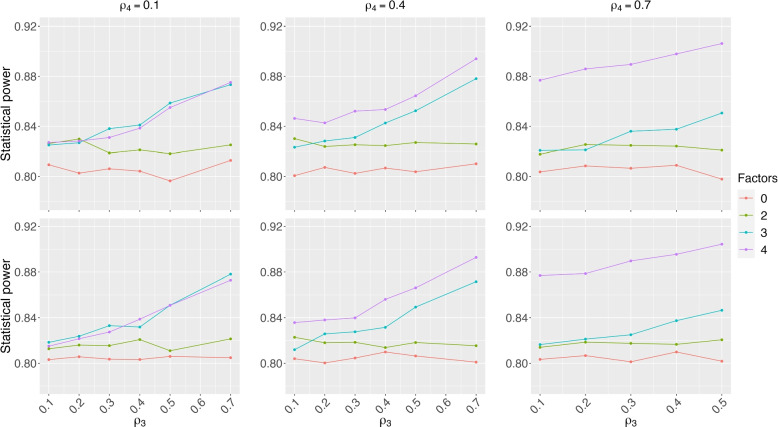
Statistical power of a two-arm CAR design as a function of $\rho _3$, given $\rho _4$ from 0.1 to 0.7. In the CAR design, the treatment assignment probability was set as 60%. The first and the second row has the correlation $\rho _2=0.2$ and 0.1. When the number of factor is zero, it is a CR design

In Fig. 6, the statistical power was presented as a function of $\rho _3$ which is the correlation between outcome and the third factor, with $\rho _4$ from 0.1 to 0.7. The correlation between the multinomial factor and the outcome was assumed to be $\rho _2=$0.2 (the first row) and 0.1 (the second row). We calculated the statistical power by using the PS_p=0.6_ with 2, 3, and 4 factors as covariates. The simulated power is very close to the nominal level of 80% for the CR. When correlation is low (e.g., $\rho _3=\rho _4=$10%), the statistical power of CAR designs is similar to that of CR. As correlation goes up ($\rho _4$ from 0.1 to 0.7), the statistical power could be increased by more than 10% with the simulated statistical power being above 90%.

The statistical power gain using the CAR design with two factors can consistently improve more than 2% as compared to the CR design. The statistical power of the CAR designs including 3 factors or more is an increasing function of $\rho _3$. When $\rho _3$ goes up to 0.7, the statistical power could be above 90% at the nominal level of 80%. When $\rho _4$ is small (e.g., $\rho _4=0.1$), the statistical power gain is very limited by adding the fourth factor to the model with 3 factors. However, when $\rho _4$ is medium to large (e.g., 0.4, 0.7), it may further increase the statistical power by including that additional factor in the CAR design. We observe similar findings in comparing statistical power based on PS_p=0.6_ with 2, 3, and 4 factors, as a function of $\rho _3$.

**Fig. 7:**
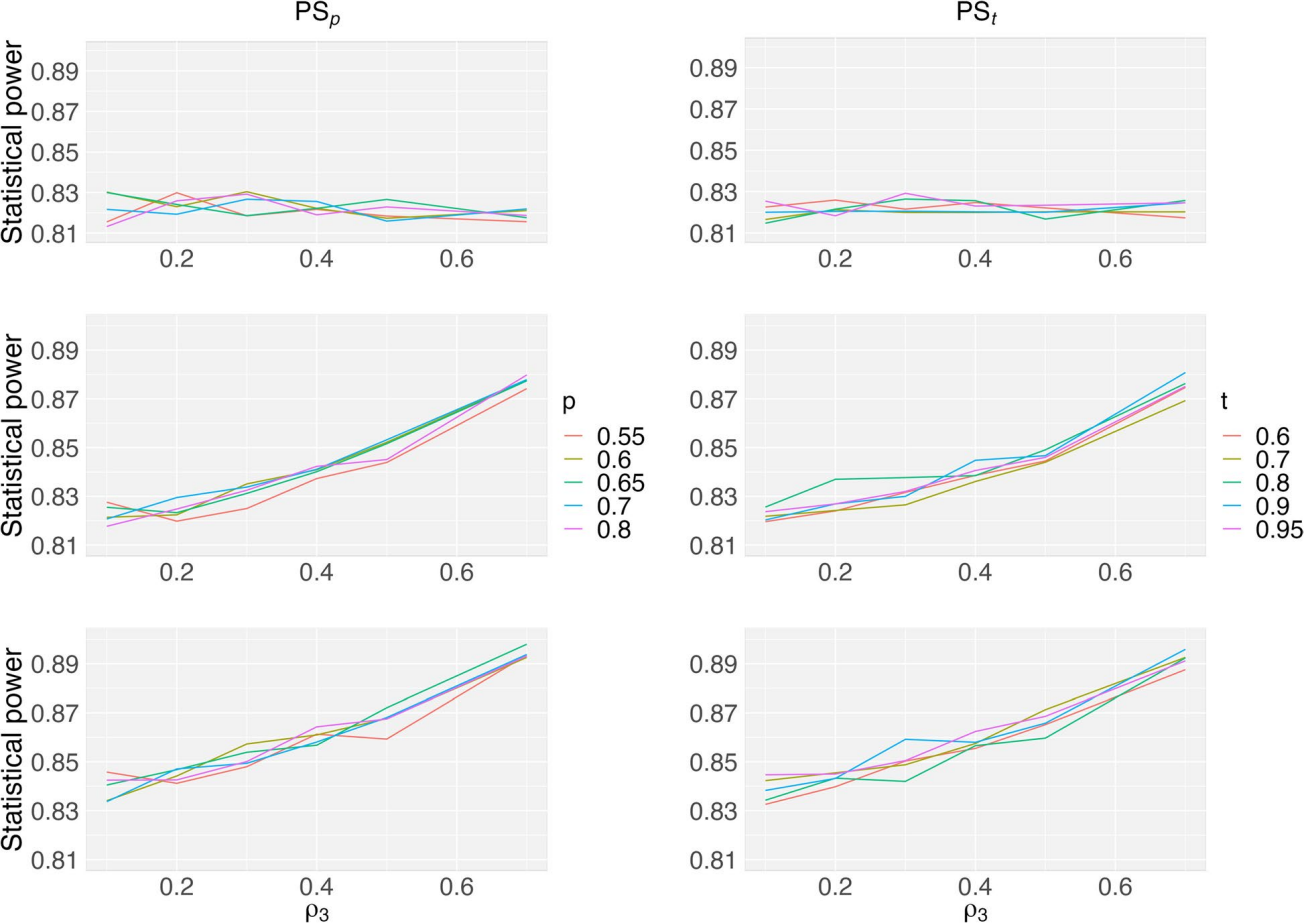
Statistical power of a two-arm CAR design as a function of $\rho _3$, given $\rho _1=0.3$, $\rho _2=0.2$, and $\rho _4=0.4$. Five different *p* values (0.55 to 0.8) in the PS_p_ method and five *t* values (0.6 to 0.95) in the PS_t_ method were studied, for two, three, and four factors (row 1, row 2, and row 3)

We further compare the statistical power as *p* in the PS_p_ method and *t* in the PS_t_ method go up in Fig. 7. The statistical power was plotted as a function of $\rho _3$ given $\rho _1=0.3$, $\rho _2=0.2$, and $\rho _4=0.4$. Five different *p* values (0.55 to 0.8) and 5 *t* values (0.6 to 0.95) were studied. When two factors are used in CAR designs (first row), the statistical power is not sensitive to the value of $\rho _3$. When we have three or four factors (the middle row, and the bottom row), the statistical power is an increasing function of $\rho _3$. When $\rho _3=0.4$, the statistical power may be increased by 4% when two additional factors are added to a model having two existing factors. For the PS_p_ method, the *p* values between 0.6 to 0.7 have good performance with regards to the statistical power in many configurations. For the PS_t_ method, a high value of *t* (e.g., 0.8 or above) is associated with a large statistical power. Given the $\rho _3$ value and the number of factors, the statistical power difference between these considered methods is small which is around 2%.

### An example

We used data from the Second Multi-centre Intra-pleural Sepsis Trial (MIST2) to illustrate the application of the CAR designs [42, 43]. The MIST2 trial was a four-arm randomized trial to investigate the efficacy of intrapleural tissue plasminogen activator (t-PA) and DNase for patients with pleural infection. The primary outcome was the change in pleural opacity at day 7 from day 1. We used the estimated correlation between the primary outcome and three categorical factors: hospital-acquired infection ($\rho _1=0.12$), large tube size ($\rho _2=0.16$), and drain present ($\rho _3=0.27$). These correlation coefficients were presented in the article by Kahan et al. [43].

Suppose we are going to compare a new treatment with the gold standard: a randomized two-arm study. To detect a medium effect size of 0.39 in an early phase trial, the sample size per group is estimated as 105 (a total of 210 patients) based on the two-sample t-test. Given that sample size, the simulated statistical power from 20,000 simulations is 79.95% in the statistical model without controlling for other covariates. For the CAR designs, the statistical power is between 81.2% and 82.2% when these three factors are considered in the randomization and adjusted in the primary analyses. The highest power is obtained when the PS_q=0.63_ approach in conjunction with the SD method in calculating the imbalance score is used in the CAR design.

## Discussion

From simulation studies, we found that the increase in statistical power depends on the correlation between covariates and the outcome. Specifically, adjusting for covariates that are strongly correlated with the outcome leads to a greater reduction in the standard errors for the treatment effect, and therefore a larger increase in the statistical power [44]. When adjusting for key confounders in the context of binary or survival outcomes though, it is expected that the standard errors would increase. However, this can be counterbalanced by an increase in the estimated treatment effect, which ultimately contributes to enhanced power [45–48]. It should be noted that in the current study, our focus was exclusively on continuous outcomes. However, binary and survival outcomes represent areas of significant interest that have not been explored in this work. These outcome types could serve as a focus for future research endeavors.

Additionally, when we compared the statistical power between CAR designs and CR designs, there were some methodological disparities that merited attention as we implemented different strategies for covariate control. Specifically, we did not adjust for any covariates when analyzing the statistical power of CR designs, whereas for the CAR designs, we did control for a varying number of prognostic factors. This discrepancy introduces a layer of complication to the interpretation of the results, as it becomes challenging to determine the exact effect of the CAR methods on the observed increase in statistical power. It raises the question of whether the power increase is genuinely attributable to the effectiveness of CAR designs, or if it is merely the result of the inclusion of additional covariates in the statistical model. Therefore, discerning the specific contribution of CAR methods to the improvement of statistical power remains an intricate issue. Future research could compare the statistical power by conducting identical data analyses while varying only the randomization methods employed. This would provide a clearer understanding of the influence of different randomization techniques on the resulting statistical power.

## Data Availability

Not applicable. This is a manuscript to develop novel statistical approaches, therefore, no real data is involved.
